# Optimized submerged batch fermentation strategy for systems scale studies of metabolic switching in *Streptomyces coelicolor* A3(2)

**DOI:** 10.1186/1752-0509-6-59

**Published:** 2012-06-07

**Authors:** Alexander Wentzel, Per Bruheim, Anders Øverby, Øyvind M Jakobsen, Håvard Sletta, Walid A M Omara, David A Hodgson, Trond E Ellingsen

**Affiliations:** 1 Department of Biotechnology, SINTEF Materials and Chemistry, 7465, Trondheim, Norway; 2 Department of Biotechnology, Norwegian University of Science and Technology (NTNU), 7491, Trondheim, Norway; 3 School of Life Sciences, University of Warwick, Gibbet Hill Road, Coventry, CV4 7AL, UK

## Abstract

**Background:**

Systems biology approaches to study metabolic switching in *Streptomyces coelicolor* A3(2) depend on cultivation conditions ensuring high reproducibility and distinct phases of culture growth and secondary metabolite production. In addition, biomass concentrations must be sufficiently high to allow for extensive time-series sampling before occurrence of a given nutrient depletion for transition triggering. The present study describes for the first time the development of a dedicated optimized submerged batch fermentation strategy as the basis for highly time-resolved systems biology studies of metabolic switching in *S. coelicolor* A3(2).

**Results:**

By a step-wise approach, cultivation conditions and two fully defined cultivation media were developed and evaluated using strain M145 of *S. coelicolor* A3(2), providing a high degree of cultivation reproducibility and enabling reliable studies of the effect of phosphate depletion and L-glutamate depletion on the metabolic transition to antibiotic production phase. Interestingly, both of the two carbon sources provided, D-glucose and L-glutamate, were found to be necessary in order to maintain high growth rates and prevent secondary metabolite production before nutrient depletion. Comparative analysis of batch cultivations with (i) both L-glutamate and D-glucose in excess, (ii) L-glutamate depletion and D-glucose in excess, (iii) L-glutamate as the sole source of carbon and (iv) D-glucose as the sole source of carbon, reveal a complex interplay of the two carbon sources in the bacterium's central carbon metabolism.

**Conclusions:**

The present study presents for the first time a dedicated cultivation strategy fulfilling the requirements for systems biology studies of metabolic switching in *S. coelicolor* A3(2). Key results from labelling and cultivation experiments on either or both of the two carbon sources provided indicate that in the presence of D-glucose, L-glutamate was the preferred carbon source, while D-glucose alone appeared incapable of maintaining culture growth, likely due to a metabolic bottleneck at the oxidation of pyruvate to acetyl-CoA.

## Background

*Streptomyces coelicolor* A3(2) is the best studied member of the genus *Streptomyces*[1], which provides the source of numerous antibiotic compounds in clinical use today. The genome sequence of *S. coelicolor* A3(2) was published in 2002 [2] revealing its genome as one of the largest bacterial genomes known to date. Like most members of the genus, it exhibits a complex life-cycle including the differentiation of substrate mycelium to aerial mycelium and the formation of spores [3]. Upon nutrient limitation, *S. coelicolor* A3(2) responds with cellular differentiation, growth cessation and with its substrate mycelium subsequently initiating production of secondary metabolites [4,5]. These include among others calcium-dependent antibiotic (CDA, [6]) and the coloured actinorhodins (Act, [7]) and prodiginines (e.g. undecylprodigiosin, RED, [8]). The regulatory events taking place during the transition from primary to secondary metabolic phase are complex and involve a plethora of both pleiotropic and pathway-specific regulators most likely linked together in a complex regulatory network [9]. A lot of effort has been put into the identification and characterization of individual components of the regulatory network and linkages within. Nevertheless, many linkages and especially the involvement of yet unidentified components of the network still remain cryptic, requiring novel approaches of global and multi-layered analysis of metabolism. Results from systems level studies have the potential to provide a highly detailed global understanding of the events occurring during transition from primary to secondary metabolism [10]. Such advances are likely to make an important contribution to the identification of possible new handles for transition triggering and ultimately to vital biotechnological improvements of antibiotic production.

A systems biology approach to globally study metabolic switching in *S. coelicolor* A3(2) would consist of iterative cycles of (i) cultivation, (ii) highly time-resolved data generation covering the different accessible levels of metabolism (transcriptome, proteome, metabolome), and (iii) mathematical network modelling. However, such an approach requires the cultivation system, providing biomass for all types of metabolic analysis, to be of excellent quality and reliability. Important features of a suitable submerged batch fermentation system are to provide (i) a sufficiently high biomass concentration to allow for extensive highly time-resolved sampling for all kinds of subsequent analysis, from many hours before the event of nutrient depletion to long into secondary metabolite production phase, (ii) an excellent reproducibility in biological replicas, closely monitored by applying appropriate on-line and off-line analyses, (iii) compliance of all cultivation media components with the subsequent methods of ’omics analyses, (iv) media compositions providing a single defined nutrient depletion/triggering event and (v) significantly high antibiotic production rates serving as an indication for a clear switch to production phase with accompanying high expression levels of genes involved in secondary metabolite production.

The fact that *S. coelicolor* A3(2) shows a mycelial growth habit adds to the challenges of developing a consistent cultivation method. Shear forces as a function of stirrer speed during the fermentation trials influence the mycelial pellet size. This may as a consequence have led to altered growth rates and access to nutrients and dissolved oxygen, along with an increased culture heterogeneity, all of which could have affected reproducibility. It has previously been described that high shear forces, caused by high agitation speeds, to maintain elevated levels of dissolved oxygen affected secondary metabolite production in different streptomycetes, e.g. leading to a reduction in avermectin B_1a_ production in *S. avermitilis*[11] and an improvement in clavulanic acid production in *S. clavuligerus*[12].

In the scientific literature dealing with *Streptomyces* species and in particular *S. coelicolor* A3(2) cultivations, several different media are well-documented for both shake flask and fermentation experiments [13-18]. However, only limited information is provided about the presence of a clear transition phase where nutrient depletion occurs at high biomass and well before production of the coloured antibiotics actinorhodin (Act) and prodiginines (RED) was obtained, possibly indicating substantial problems in achieving a proper transition phase.

Here, we report the development and evaluation of an optimized cultivation system for studying the metabolic switching of *S. coelicolor* A3(2) in response to phosphate or L-glutamate depletion, taking into account the special requirements of systems biology research involving mycelial bacteria such as streptomycetes. Due to the complexity of the system, it was in general considered of crucial importance to standardize all processes along the cultivation pipeline. This included: (i) the different stages of spore batch generation and characterization, (ii) the germination protocol to produce the inoculum to the parameters used in the batch fermentation culture and (iii) the protocols to withdraw and process samples for the different kinds of subsequent analyses.

One interesting feature of the optimized cultivation system is that two sources of carbon (D-glucose and L-glutamate) were required to obtain sufficient biomass concentrations before the onset of secondary metabolite production. We also show that prevention of low levels of dissolved oxygen during the cultivation exhibited a positive effect on the reproducibility of antibiotic production in this system.

Based on the cultivations and respective fermentation data included in the present study, different aspects of global metabolic switching in *S. coelicolor* A3(2) have already been studied in detail and published by us, including high resolution time-course analyses on the levels of gene transcripts [19-22], proteins [23] and metabolites [24], as well as including different mutant strains [23-25]. Additional studies based on the presented optimized fermentation strategy and the fundamental results of this study will lead to a further improvement of the molecular understanding of antibiotic production in streptomycetes.

## Methods

### Strain and general cultivation parameters

Experiments were performed using the prototrophic, plasmiD-free (SCP1^-^ SCP2^-^) strain M145 of *S. coelicolor* A3(2) [13]. Germinated spores were used as the inoculum in all cultivations. Spore batches were generated by cultivation on soy flour-mannitol (SFM) agar plates [13], harvesting by scraping of spores and suspension in 20% (v/v) glycerol, and storage in aliquots at −80°C. In the optimized inoculum preparation procedure, 10^9^ CFU of strain M145 spores [typically corresponding to approx. 1 mL of a thawed spore stock in 20% (v/v) glycerol] were germinated for 5 hours at 30°C and 250 rpm in 250 mL baffled shake-flasks with 2 g of 3 mm glass beads and 50 mL 2x YT medium [26]. The germinated spores were harvested by centrifugation (3200 x g, 15°C, 5 min) and re-suspended in 5 mL ion-free water. An even re-suspension and dispersion of the germinated spores was achieved by vortex mixing (30 s), ensuring comparable inocula among biological replicas. Each fermentor (1.8 liter growth medium) was inoculated with 4.5 mL germinated spores suspension (corresponding to 9x10^8^ CFU).

**Figure 1:**
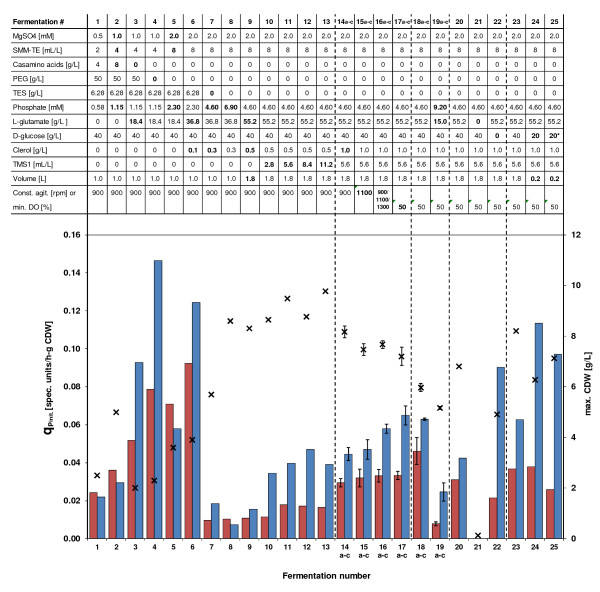
**Listing of cultivation conditions applied in (upper part), and presentation and comparative characterization of specific productivities determined (lower part) for fermentations of the present study; single fermentations runs, except for where indicated a-c (three biological replicas).** SMM-TE, trace elements solution of medium SMM [26]; PEG, polyethylene glycol 6000; TES, N-Tris(hydroxymethyl)methyl-2-aminoethanesulfonic acid; TMS1, trace mineral solution 1; const. agit. or min. DO, cultivation performed at given constant agitation from the start/culture started at min. agitation (~325 rpm) or agitation was adjusted automatically to maintain at least the given percentage of dissolved oxygen (DO); 900/1100/1300 (ferm. #16a-c) indicates manual change of constant agitation from 900 to 1100 to 1300 rpm after 21 and 26 h, respectively, in each of three biological replicas to prevent a DO of lower than approx. 50%, returned to 900 rpm after 45 h. Ferm. #18a-c and #19a-c were experiments with massive volume reduction due to high resolution time-series sampling. Ferm. #21, L-glutamate amount exchanged with ammonium sulfate of the respective molar amount of nitrogen; ferm. #24/25, use of custom-made 350 mL fermentation vessels with 200 mL working volume; ferm. #25, use of 20 g/L fully ^13^ C-labelled D-glucose (*). All fermentations were performed at 30°C and pH 7.0, automatically adjusted with 2 M HCl and 2 M NaOH. Initial specific productivity q_Pinit_ of secondary metabolites determined by measuring the pigment levels in culture samples using the RED/TBP assays and plotting the data as a function of time, linear regression of the initial production data points after production start (usually within the first 15 h after sec. metabolites have first been detected); max CDW, maximum biomass concentration (cell dry weight). X symbols indicate the maximum biomass concentration, red and blue bars the initial specific productivity of RED and TBP, respectively. Bold font indicates changes in cultivation conditions in the order of presentation from ferm. #1 to ferm. #25.

Cultivations were performed in 3-liter fermentors (Applikon) with an initial culture volume of 1 L or 1.8 L. All media were based on ion-free water, and all chemicals used were of analytical grade. An overview of all fermentations and respective media specifically referred to in this study are summarized in Figure 1. The optimized growth medium used for studying the effect of phosphate depletion during batch fermentation (SSBM-P) has been reported before [19] and consisted of Na-glutamate, 55.2 g/L; D-glucose, 40 g/L; MgSO_4_, 2.0 mM; phosphate, 4.6 mM; supplemented minimal medium trace element solution SMM-TE [26], 8 mL/L and TMS1, 5.6 mL/L. TMS1 consisted of FeSO_4_.7 H_2_O, 5 g/L; CuSO_4_.5 H_2_O, 390 mg/L; ZnSO_4_.7 H_2_O, 440 mg/L; MnSO_4_.H_2_O, 150 mg/L; Na_2_MoO_4_.2 H_2_O, 10 mg/L; CoCl_2_.6 H_2_O, 20 mg/L, and HCl, 50 mL/L. The optimized medium for studying the effect of L-glutamate depletion (SSBM-E) was identical to SSBM-P except for the concentrations of Na-glutamate and phosphate adjusted to be 15 g/L and 9.2 mM, respectively. Clerol FBA 622 fermentation defoamer (Diamond Shamrock Scandinavia) was added to the growth medium before inoculation as given in Figure 1. The presence of up to 1 g/L of the antifoam agent did not have a significant influence on biomass yield and actinorhodin and undecylprodigiosin productivities. Neither did the omission of TES buffering agent, originally present in the source medium of the media development process. Throughout fermentation trials, pH 7.0 was maintained constant by automatic addition of 2 M HCl (SSBM-P and SSBM-E) and 2 M NaOH (SSBM-E after L-glutamate depletion). Dissolved oxygen levels were either left uncontrolled by choosing a constant agitation speed (900 rpm, 1100 rpm) or a stepwise manual increase/decrease of stirrer speed (900/1100/1300 rpm), or alternatively maintained at a minimum of 50% by automatic adjustment of the stirrer speed (minimal agitation 200 or 325 rpm). The aeration rate was constant 0.5 L/(L x min) sterile air. Dissolved oxygen, agitation speed and carbon dioxide evolution rate were measured and logged on-line, while samples for the determination of cell dry weight and levels of growth medium components and secondary metabolites concentrations were withdrawn throughout the fermentation trials.

Shake flask experiments were performed at 30°C and 250 rpm in 250 mL baffled shake flasks using 40 mL medium containing 5.52 mM phosphate, 0.6 mM MgSO_4_, 2.4 mL/L SMM-TE, 50 g/L PEG6000 and 10 g/L MOPS, pH 7.0 and the carbon and nitrogen source combinations as given in Additional file 1: Table S1. Germinated spores (5x10^8^ CFU per litre medium) produced as described above were used as an inoculum. Growth was followed by OD_450_ measurements and production of secondary metabolites by visual inspection.

### Determination of concentrations of secondary metabolites and media components

Levels of phosphate, D-glucose, ammonium and L-glutamate were measured spectrophotometrically by using the SpectroQuant Phosphate test kit, the Lactose/D-glucose test kit, the Ammonium test kit (all R-Biopharm), and the L-glutamate Bioassay kit (US Biological), respectively, following the manufacturer’s instructions after downscaling to 96-well plate format. Undecylprodigiosin (hereafter and throughout the text referred to as 'RED') levels were determined spectrophotometrically at 530 nm after acidified methanol extraction from the mycelium [27]. To determine the amount of total blue pigments (actinorhodins; hereafter and throughout the text referred to as 'TBP'), cell culture samples were treated with KOH (final concentration 1 M) and centrifuged, and the absorbance of the supernatants at 640 nm was determined [27]. γ-actinorhodin (hereafter and throughout the text referred to as 'γ-Act') in culture supernatants was quantified spectrophotometrically in 0.01 M NaOH (final concentration) at 608 nm [27].

### ^13^ C labelling enrichment and metabolite analysis

For metabolite analysis, two 200 mL scale cultivations in 350 mL custom-made mini-fermentors were performed using SSBM-P medium and cultivation conditions similar to the 1.8 L cultivations described above. Exceptions were an increased minimal agitation of 545 rpm and the use of a reduced but still excess amount of 20 g/L D-glucose. In one of the two parallels, ^13^ C_6_D-glucose (Sigma, 389374) was used instead of non-labeled D-glucose. For metabolite analysis sampling, 5 mL culture was applied to a 0.8 μm Supor800 (Pall) filter placed on a funnel on a vacuum manifold and immediately washed twice with slightly hypertonic 2.63% (w/v) NaCl solution before the filter was transferred to 25 mL 60% (v/v) methanol, pre-cooled on an ethanol bath at −23°C. The time between sampling and quenching in cold methanol was 10–15 s. Four identical samples were taken at each of two sampling points (one in growth phase and one in stationary/antibiotic production phase). Samples were stored at −80°C. Metabolite extraction was performed at low temperature by subjecting the samples to three subsequent freeze-thaw cycles on liquid nitrogen and at −23°C. After the final thawing step, samples were centrifuged for 5 min at 8000 rpm in a pre-chilled Heraeus Biofuge at −9°C. 18 mL supernatant were frozen at −80°C and freeze-dried for 24 h. Metabolites were dissolved in 0.5 M NaOH, 41.6% (v/v) methanol, 8.4% (v/v) pyridine, and derivatized in silanized glass tubes upon addition of 5% (v/v) methyl chloroformate twice while whirl-mixing for 30 s after each addition [28]. Derivatized metabolites were extracted into 0.5 vol. chloroform, and the derivatization reaction was stopped by addition of 0.5 vol. 50 mM NaHCO_3_. The organic phase was dried by addition of Na_2_SO_4_, and 2 μL were analyzed by GC-MS, performed using an Agilent 6890GC-5975MS system, operated at 70 eV and equipped with a 30 m x 250 μm x 0.25 μm Agilent 122-5532GDB-5MS + DG capillary column. The 2 μL of injected sample (pulsed splitless) was run on a 35 min temperature gradient from 45°C to 300°C. The MS was operated in scan mode (start after 6 min, mass range 50–550 a.m.u. at 2.5 scans/s). Metabolites from the naturally labelled D-glucose cultivation were identified using Agilent ChemStation DRS (Deconvolution Reporting Software) and AMDIS (NIST) software. The method used refers to a database consisting of 106 compound peaks including those of 32 organic acids and amino acids. Subsequently, analysis of GC-MS chromatograms of ^13^ C-enriched metabolite extracts was performed. This analysis was based on manual inspection of isotope distributions of the various ion clusters in mass spectra at the same retention times as those amino acids identified in the natural labelled cultivation. The mass isotopomer distribution of respective ion clusters of metabolites in samples from the two parallel cultivations were converted into summed fractional labelling (SFL) as described before [29]. Since MCF derivatization has not been used in metabolic flux estimation, a preliminary study was conducted to identify metabolite carbon atoms to the different ion clusters in the fragmentation mass spectra (data not shown). The data were normalized to a percentage ^13^ C-enrichment score and the SFLs of the natural labelled cultivation were subtracted in the presented data to correlate for the natural ^13^ C-abundance.

### Expression of pyruvate dehydrogenase genes with different carbon sources

Strain M145 spores were germinated as described above. Germinated spores were, to a final OD_450_ of 0.1, transferred to 50 mL minimal salts medium containing 15 mM (NH_4_)_2_SO_4_, 50 g/L Polyethylene glycol 6000, 2.5 mM MgSO_4_.7H_2_O, 1 mM NaH_2_PO_4_/K_2_HPO_4_ buffer pH 7.2, 0.01% (^w^/_v_) antifoam 204, 25 mM BisTrisPropane pH 7.2, supplemented minimal medium trace element solution 8 mL/L SMM-TE and 5.6 mL/L TMS1 and 50 mM carbon source (i.e. arabinose, alanine, aspartate, glucose + glutamate, glucose, glutamate, proline, Tween-20, Tween-40, Tween-60, Tween-80, xylose) in stainless steel spring baffled 500 mL Erlenmeyer flasks. Flasks were incubated at 30°C with shaking at 250 rpm and biomass collected by centrifugation at mid-exponential phase (after approximately 5–6 doublings). RNA was extracted and applied to Affimetrix arrays as described in Nieselt *et al.*[19]. Data were handled as in Nieselt *et al.*[19] using GeneSpring®.

## Results

### Semi-defined SMM derived media provide a clear transition phase at biomasses sufficiently high for full-scale ‘omics sampling

Our first focus was on the development of a fully defined medium that provided sufficient biomass concentrations of strain M145 (>4 g/L CDW) for highly time-resolved sampling series for full-scale ‘omics analysis (transcriptome, proteome, metabolome) already several hours before the depletion of one specific nutrient followed by a clear transition to antibiotic production phase. Several well documented growth media were tested in a 1 L fermentation scale using germinated spores as an inoculum: Evans medium [15] with either ammonium or nitrate as the sole nitrogen source, AMM [17], *Streptomyces* Minimal Medium [16], NMMB [18] and SMM [30]. However, neither of the fully defined media provided fast growth but rather led to production of antibiotics starting at low biomass concentrations (data not shown). The most promising result was obtained for the semi-defined SMM medium (Figure 1, ferm. #1), containing casamino acids as a complex supplement. This medium was therefore chosen for the subsequent media development process. For SMM, rapid growth up to a biomass concentration of 2.5 g/L and initial specific productivities of RED and TBP of >0.02 spec. units/(h x g/L CDW) were obtained (Figure 1). Strauch *et al.* (1991) developed the SMM medium to give reproducible antibiotic production in a semi-defined medium to study the role of the stringent response [30]. The medium contained enough casamino acids to promote rapid growth. Upon depletion of the growth limiting component in the complex medium, stationary phase and antibiotic production were induced. This reproducible antibiotic production was only seen with strain M145, the engineered plasmid free prototroph also used in the present study. Other prototrophic *S. coelicolor* strains often give more rapid primary growth rates but do not show reproducible antibiotic production (Hodgson, DA, unpublished). In ferm. #1, antibiotic production was first detected a few hours after depletion of phosphate in the medium. The biomass concentration could be proportionally increased to almost 5 g/L CDW by doubling the amounts of phosphate, MgSO_4_, SMM trace elements solution SMM-TE (see Materials and Methods section for composition) and casamino acids (ferm. #2), exhibiting a small positive impact on the specific productivities of RED and TBP.

### Substitution of casamino acids with L-glutamate leads to a fully defined cultivation medium

In SMM, casamino acids are the sole source of nitrogen comprised of amino acids in different concentrations. Dependent on the metabolic usability of the individual components in this complex mixture, constant metabolic shifting can be expected to occur from successive depletion of the respective preferred amino acid in the pool. The molecular consequences of this would be difficult to interpret and are likely to interfere with the aim of studying the effect of one single defined nutrient depletion event. We therefore systematically evaluated the possibility to substitute casamino acids in the medium with nitrogen from defined, preferentially inorganic compounds. In a series of shake flask experiments, ammonium, urea and also L-glutamate in combination with different organic carbon sources were tested (see Additional file 1: Table S1). In all ammonium and urea based cultures, slow or no growth were observed, and/or antibiotic production started early during growth phase at very low biomass concentrations. Only the use of nitrogen from the amino acid L-glutamate or casamino acids resulted in good growth. The desired combination of a fully defined medium, a fast growth phenotype and a late start of secondary metabolite production were only obtained for L-glutamate in combination with D-glucose or glycerol indicating this amino acid as an excellent source of nitrogen for strain M145. L-glutamate at a similar molar nitrogen concentration as casamino acids used in ferm. #2, in combination with D-glucose, proved to be a good defined substitute for the mixed nitrogen source (ferm. #3). A clear transition phase was obtained, and productivities of both TBP and RED were increased (Figure 1). However, biomass yield decreased to about 2 g/L CDW due to lower total amounts of phosphate present in the medium as the casamino acids mixture demonstrably contained a significant amount of inorganic phosphate.

Removal of PEG from the medium used in ferm. #3, another complex component in the initial medium added to control morphology and not compatible with downstream mass spectrometry-based analytical methods (proteome and metabolome analysis), was well tolerated, leading to increased productivity of TBP and RED at a maintained biomass concentration (ferm. #4). Microscopy revealed, as expected, the mycelial pellets being slightly larger in size in the absence of PEG (data not shown). By doubling the amounts of phosphate, SMM-TE and MgSO_4_, the biomass yield could be increased to almost 4 g/L CDW in the L-glutamate based medium, though resulting in a significant decrease in productivity of TBP, but not RED (ferm. #5).

Doubling the amount of L-glutamate at this point restored the previously obtained productivities (ferm. #6). The biomass concentration could be increased to more than 8 g/L CDW by increasing the phosphate concentration from 2.3 mM (ferm. #6) to 4.6 mM (ferm. #7) and further to 6.9 mM (ferm. #8), indicating that the amount of phosphate in these media was the growth limiting component. However, after depletion of the increased amounts of phosphate, productivities of TBP and RED obtained were dramatically reduced indicating not only the importance of the concentration of phosphate in the medium for TBP and RED production, but also its ratio to other nutrients. This finding may be explained by possible phosphate repression, observed for quite a number of antibiotics at excess phosphate concentrations [31,32]. In these studies, upon provision of excess phosphate, production start was delayed, while addition of calcium ions to sequester phosphate triggered production. To minimize the potential effect of phosphate repression, the lower concentration of 4.6 mM phosphate was chosen for further media development as sufficient concentrations of biomass (approx. 6 g/L) were produced already from this concentration. At this point, an increase in the L-glutamate concentration to 55 g/L ensured that L-glutamate remained in excess until 90 h after inoculation (ferm. #9).

### TBP and RED productivities were optimized by addition of trace metal solution TMS1

In subsequent fermentation experiments to increase TBP and RED productivity, we introduced increasing amounts of trace metal ions in the culture (ferm. #10-13). The optimal amount of trace mineral solution TMS1, for the production of TBP and RED was achieved with addition of 5.6 mL/L. TMS1 provides iron as the main component, leading to a total concentration of 104 μM iron in media containing 5.6 mL/L TMS1. The maximum biomass concentration was found to be not affected by varying the addition of TMS1, indicating phosphate depletion in this medium being the sole trigger for switching to secondary metabolite production phase. It has been reported before that actinorhodin production is influenced by media iron concentrations [33]. However, documentation of iron regulation of secondary metabolism in streptomycetes is scarce, and dependent on media used and other cultivation parameters applied, both repression and stimulation of secondary metabolite production by iron ions have been reported [33-36].

### Prevention of low dissolved oxygen levels increased reproducibility of culture growth and secondary metabolite production using the phosphate depletion medium SSBM-P

The fully-defined medium finalised in the 1.8 L scale in fermentation #11 fulfils the requirements of a clear transition phase in response to phosphate depletion as the only trigger for metabolic switching at a biomass concentration suitable for highly repetitive time-series sampling for advanced analysis of transcripts, proteins and metabolites. However, low levels of dissolved oxygen down to almost 10% were regularly detected by us in fermentations starting with 4.6 mM phosphate in the 1.8 L scale and using a constant agitation of 900 rpm, possibly representing an important source of variation in antibiotic production and the preceding regulatory events.

From the scientific literature it is clear that antibiotic production by streptomycetes itself can be affected by the concentration of dissolved oxygen. For example, clavulanic acid production titres and rates by *S. clavuligerus* were found to vary significantly when different fixed stirrer speeds or a constant 50% air saturation were applied in the 4 L scale using complex medium [12], and the maintenance of a minimum DO level of 40% led to an optimized production of avermectin B_1a_ by *S. avermitilis*[11]. However, the authors of both references also report that high shear forces as a consequence of high agitation speeds to maintain a given DO level can affect antibiotic production yields. Here, the effects seems to be the result of a complex interplay with these and other parameters such as media composition and pre-culture conditions and can be either negative or positive dependent on the respective strain. Though the influence of DO levels and shear forces on antibiotic production in several streptomycetes has been reported, the evaluation and optimization of the reproducibility to meet the requirements of systems scale studies has, to our knowledge, never been the subject of interest.

In three biological replicas cultivated in parallel at a constant agitation of 900 rpm (ferm. #14a-c), we observed that the levels of dissolved oxygen dropped to 13% and stayed below 40% for as long as 21 h, resulting in an average standard variation of 17% of the TBP levels; see Table 1, where the average standard variation is calculated as an average value of the standard deviation of the TBP levels between the three biological replicas at the different time-points in the production phase between 43 and 79 h of cultivation. In an attempt to reduce the extended period of low DO levels, the constant stirrer speed was increased to 1100 rpm (ferm. #15a-c). However, this resulted in similarly low DO minimum levels (12%) as observed for 900 rpm (ferm. #14a-c), though DO levels returned to >40% faster than at the lower stirrer speed, significantly reducing the time interval of low DO (Table 1). The specific productivities of RED and TBP at 1100 rpm were not significantly changed compared to 900 rpm (Figure 1). However, microscopic analysis revealed that higher constant agitation speeds from the beginning led to smaller average mycelial pellet size in response to the increased shear forces applied. In addition, in such cultures an earlier depletion of phosphate in the medium was obtained indicating an increased number of pellet forming units caused by mechanical fragmentation of the mycelium (data not shown). In order to entirely prevent DO levels below 40%, in the run of three parallel fermentations, agitation was increased manually from 900 rpm to 1100 rpm after 21 h and further to 1300 rpm after 27 h, later being reduced again to 900 rpm 44 h after inoculation (ferm. #16a-c). Again, productivity of RED was not affected, while TBP productivity increased slightly (Figure 1). However, by ensuring relatively high DO levels throughout the cultivation, the average standard deviation of TBP levels between the three biological replicas was significantly reduced to 6%, while the average standard deviation of γ-actinorhodin levels still remained above 10% (Table 1).

**Table 1:** Production and reproducibility in response to different agitation regimes

Ferm. #	Agitation regime					production lag time			q_Pinit_			Average standard		
		DOmin	t(<40% DO)	μmax	max CDW	[h]			[spec. units/g CDW-h]			deviation [%]		
		[%]	[h]	[h-1]	[g/L]	Red	γ–Act	TBP	Red	γ–Act	TBP	Red	γ–Act	TBP
14a-c	900 rpm	12.7	21.2	0.275	8.17	9.2	17.5	18.7	0.030	0.008	0.045	5.8	18.8	17.3
		2.1	1.0	0.010	0.23	0.3	1.5	1.8	0.002	0.001	0.003			
15a-c	1100 rpm	12.0	12.2	0.298	7.47	11.0	13.7	14.7	0.032	0.010	0.047	6.5	12.3	11.0
		1.0	1.0	0.035	0.23	1.5	0.6	0.6	0.005	0.001	0.005			
16a-c	900/1100/	38.7	0.7	0.260	7.67	5.3	12.7	13.8	0.033	0.013	0.058	7.3	11.3	5.9
	1300 rpm	8.1	1.2	0.008	0.15	1.2	0.6	0.3	0.003	0.001	0.002					
17a-c	50% DO	50.0		0.254	7.20	5.8	13.2	14.7	0.033	0.013	0.065	5.3	1.5	4.5		
		0.0	n.a.	0.017	0.36	0.8	0.3	0.6	0.002	0.001	0.005					

Reproducibility of determined growth and production parameters in batch fermentations applying different agitation regimes; average values (large font, top) and standard deviations (small italic font, bottom) determined for three biological replicas each. DOmin, minimal dissolved oxygen (DO) during the fermentation run; t(<40% DO), time period where DO was below 40%; estim. μ_max_, maximum growth rate estimated from the exponential course of the CO_2_ evolution rate curve between 5 h and 22 h after inoculation; max CDW, maximum cell dry weight; production lag time, time period from phosphate depletion in the medium until pigments were first detected in the culture using the Red/γ-Act/TBP assays; q_Pinit_, initial specific productivity of secondary metabolites determined by measuring the pigment levels in culture samples using the RED/TBP assays and plotting the data as a function of time, linear regression of the initial production data points after production start; average standard deviation, indication of the variance between the biological replicas for the given analyses (calculated as an average value of the standard deviation between three biological replicas based on measurements at all time-points in secondary metabolite production phase (43 h to 79 h), except for those with lower than 10% of the respective maximum value). Cultivations were performed at the given constant agitation from the start, or started at min. agitation (~325 rpm), adjusted automatically to maintain at least 50% DO; 9/11/1300 rpm indicates manual change of constant agitation from 900 to 1100 to 1300 rpm after 21 and 26 h, respectively, to prevent a DO of lower than approx. 50%, agitation was returned to 900 rpm after 45 h.

In order to exclude unnecessary high agitation/shear forces and sudden condition changes, we also applied an agitation regime where stirrer speed was adjusted automatically to maintain levels of minimum 50% DO with 325 rpm stirrer speed set as the minimum agitation from the beginning (ferm. #17a-c). This resulted in a further improved TBP productivity compared to constant agitation, while again, RED productivity was not affected (Figure 1 and Table 1). Moreover, average standard deviation of RED, TBP and γ-Act detection in the biological replicas was dramatically reduced to 5%, 2% and 5%, respectively, (Table 1), rendering this strategy most suitable for the highest possible reproducibility. Maintaining a DO level above 40% by either a step-wise adjustment of stirrer speed (ferm. 16a-c) or by automatic adjustment of agitation to maintain 50% DO (ferm. #17a-c) resulted in a reduction of the time interval between phosphate depletion and the detection of RED in the medium. This reduced production lag time indicated that the DO level of cultures of strain M145 may have an important effect on events preceding production start of prodiginines (Table 1). The complex interplay between agitation speed affecting both the mycelial pellet size and the DO level and its influence on metabolic switching and antibiotic production in batch fermentations of strain M145 is currently subject to further investigations.

### Cultivations using medium SSBM-P fulfil the requirements of systems biology approaches

**Figure 2:**
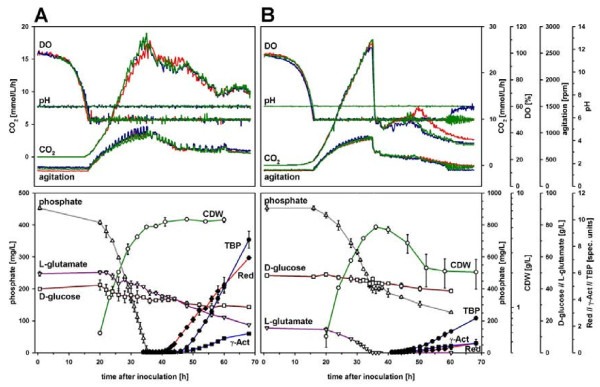
**On-line and off-line measurements as a function of time of strain M145 grown on (A) phosphate and (B) L-glutamate depletion medium.** Upper panels: on-line measurements of three individual biological replicas, lower panels: average and standard deviations of off-line analyses obtained for three biological replicas. A, ferm. #18a-c; B, ferm. #19a-c. Results in (**A**) include data identical to those published by us before [19].

The phosphate depletion medium SSBM-P in combination with the optimized cultivation conditions with extensive sampling has been introduced by us as part of a technical platform for generating reproducible expression data from *Streptomyces coelicolor* A3(2) batch cultivations [19-22]. The results of the cultivations of the three biological replicas (ferm. #18a-c) are given in Figure 2A. Both the results from on-line monitoring and from the off-line analyses indicate a very good reproducibility, and the productivities from RED, γ-Act and TBP resemble largely those of fermentations #17a-c (Figure 1). Medium SSBM-P is fully defined, and during a standard cultivation experiment of 70 h, phosphate was depleted around 35 h after inoculation, triggering a transition phase of approx. 6 h that exited into a distinct production phase with specific productivities of RED and TBP indicating efficient metabolic switching. The system therefore complies with the requirements for a cultivation system for reliable biomass production for system scale studies of metabolic switching in *S. coelicolor* A3(2) as described above and recently reported by us in studies on the molecular levels of gene transcripts [19,22], proteins [23] and metabolites [24]. However, the apparent maximum biomass concentration in the experiment with extensive repeated sampling and high volume reduction (ferm. #18a-c) was found to be slightly lower than in comparable cultivations with lower volume reduction due to more restricted sampling (ferm. #17a-c). One reason for that might lie in a larger amount of biomass deposited on the wall of the fermentor vessel observed in ferm. #18a-c. These deposits have later repeatedly been observed especially in fermentations with similar sampling regimes (unpublished data). Attachment to the vessel wall also seems to be dependent on the chosen conditions. In experiments involving medium SSBM-E (see below), a significant decrease in culture biomass concentrations has been observed after the event of L-glutamate depletion. This could be attributed to the attachment of biomass to the glass wall of the fermentor vessel and here occurred in even greater amounts than when medium SSBM-P was used. Coating the fermentor vessel by silanization did not significantly prevent attachment of mycelium to the glass vessel (data not shown) and was therefore dismissed. Nevertheless, biomass concentrations of about 5 g/L at the time of nutrient depletion allowed for repeated full-scale ‘omics sampling around and after transition phase, sufficient for studying metabolic switching events at a high time resolution.

Increase of phosphate and reduction in L-glutamate concentration converted the SSBM-P medium into a medium for studying L-glutamate depletion as an alternative trigger for metabolic switching (SSBM-E).

Decrease of L-glutamate concentration from 55.2 g/L present in SSBM-P medium to 15 g/L resulted in a sharp drop in respiration after about 35 hours after inoculation (Figure 2B). At this point, L-glutamate was found to be depleted from the medium. Nevertheless, also the phosphate concentration reached a critical level and was depleted only a few hours later (data not shown). In order to prevent low phosphate levels as an additional trigger of metabolic switching, the concentration of phosphate was doubled to 9.2 mM from the beginning providing a non-critical phosphate concentration of more than 400 mg/L at the event of L-glutamate depletion with phosphate depletion not occurring until the end of the experiment.

Similar to fermentations #18a-c using medium SSBM-P, three biological replicas were used in an analogous experiment with high resolution sampling using medium SSBM-E (ferm. #19a-c). Cultivations turned out to be almost identical replicas until L-glutamate depletion at 35 h after inoculation (Figure 2B). At this time, a maximum of 5 g/L CDW was reached, subsequently decreasing 60% to 2 g/L after approx. 50 h of the cultivation with a significant variation between biological replicas. This decrease was largely attributable to the successive attachment of biomass to the vessel wall after L-glutamate depletion as discussed above. Vessel wall attachment was likely due to a yet unknown physiological change of the mycelial pellets in response to the changed cultivation parameters. However, this effect was sufficiently low to allow for reliable sampling for ‘omics analyses until well after first appearance of RED and actinorhodins in the culture (approx. 50 h after inoculation).

Production of TBP and RED was found to be significantly lower on SSBM-E compared to SSBM-P, mirrored by strongly reduced specific productivities (Figure 1). In addition, while in ferm. #18a-c RED appeared first approx. 5 h before TBP, in ferm. #19a-c TBP appeared first and dominated until the end of the cultivations on SSBM-E. This result may indicate significantly different metabolic changes/switching events under phosphate and L-glutamate depletion conditions, respectively, in addition to the major carbon catabolism changes seen with L-glutamate depletion. Furthermore, the presence of a remaining phosphate concentration of about 4 mM at the time of L-glutamate depletion in medium SSBM-E may limit the production of RED as a consequence of phosphate repression as discussed below. Cultivations on SSBM-E have recently been shown by us to deliver high quality molecular data on different molecular levels.A metabolite profiling study involving strain M145 was performed using targeted GC-MS and LC-MS methods, providing insight into intracellular pool changes of important primary metabolites during transition phase [24]. In addition, strain M145 and a mutant strain deficient in P_II_ protein GlnK were cultivated on SSBM-E for differential transcriptome analysis at high time resolution [25]. In this study, 142 genes were identified to be differentially regulated in the two strains. However, no relevant nitrogen genes were among these, and GlnK was revealed to be not an important nitrogen sensor under the conditions tested [25].

### Fulfilling the requirements of systems biology approaches requires both D-glucose and L-glutamate to be available as carbon sources

**Figure 3:**
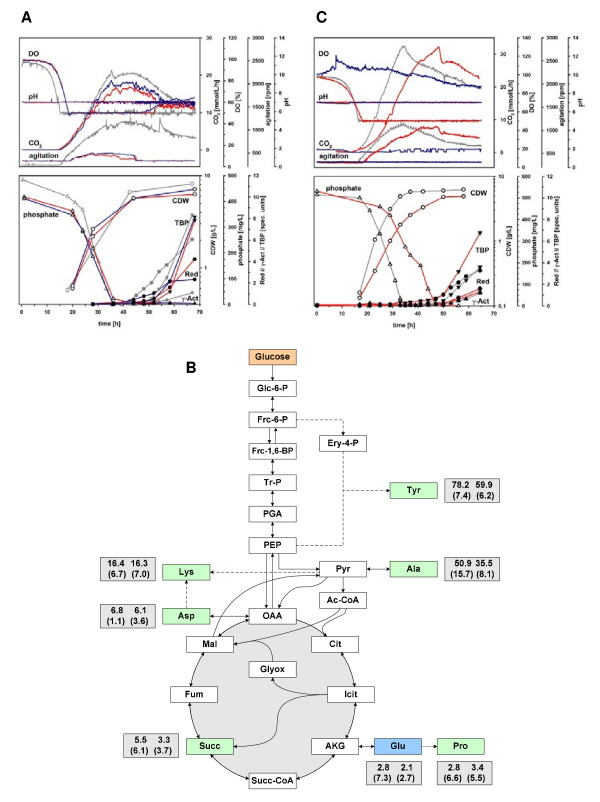
**The split carbon metabolism of M145 grown on D-glucose and/or L-glutamate.** (**A**) Summary of on-line and off-line analyses of fermentations performed in the 200 mL scale on ^13^ C_6_ D-glucose and non-labelled L-glutamate (ferm. #25, red), and on non-labelled substrates only (ferm. #24, blue), reference fermentation 1.8 L standard volume (ferm. #23, grey). (**B**) Schematic representation of parts of the central carbon metabolism including the two carbon sources used, and results from metabolite profiling and summed fractional labelling (SFL) determination (grey boxes) of selected metabolites (green/blue); Only the most significant SFLs are displayed, obtained by analysis of GC-MS data based on samples from ferm. #24 and #25 28 h (growth phase, left) and 44 h (transition phase, right) after inoculation. Relative % standard deviations are given in brackets below the SFLs. (**C**) M145 culture growth and antibiotic production in the absence of D-glucose (ferm. #22, red), or with L-glutamate (ferm. #20, grey) or ammonium (ferm. #21, blue) as the sources of nitrogen. For the latter, no significant growth was detected, not giving rise to any significant production phenotype.

The use of L-glutamate as the sole source of nitrogen and carbon led to an early onset of antibiotic production in shake flask cultures, while the substitution of L-glutamate by ammonium gave rise to a very slow or no growth phenotype ( Additional file 1: Table S1). A similar result was obtained in 1.8 L fermentation cultures using medium SSBM-P in which L-glutamate was substituted with an amount of ammonium of the same molar nitrogen content (ferm. #21). After addition of L-glutamate to the culture containing D-glucose as the sole carbon source 80 h after inoculation, respiration resumed, and biomass started to accumulate (data not shown). Compared to a reference fermentation on SSBM-P (ferm. #20), growth on an SSBM-P variant from which D-glucose was omitted (ferm. #22) was still good, though at a slightly reduced growth rate, and in addition resulted in a lower maximum biomass concentration (Figure 1, Figure 3C). In this case, TBP could be detected already as early as 32 h after inoculation, though at a relatively low specific productivity, while phosphate depletion occurred many hours later after about 46 h. Phosphate depletion in ferm. #22 still triggered the production of RED, although at a lower specific productivity than in ferm. #20, and appeared to be the main trigger for TBP production with a comparably high specific productivity. However, no clear transition phase could be obtained as was observed for the reference cultivations with D-glucose present. These observations underline the importance of L-glutamate in combination with D-glucose to provide energy and reducing power in strain M145 in order to maintain a sufficiently high growth rate to ensure distinct phases of growth, transition and production.

Clearly, there is a complicated interplay between the two carbon sources used. This can be derived from the growth rates and biomass concentrations as well as the onset and the productivities of TBP and RED production. TBP and RED differ in regard to precursor metabolites for their synthesis, the former requiring only acetyl-CoA while the latter draws acetyl-CoA, malonyl-CoA, proline, serine, glycine and the methyl group donor S-adenosylmethionine from the central metabolism [37,38]. In addition, a significant amount of energy is required for the respective synthesis, estimated to be 6 NADPH and 16 ATP for actinorhodin [39] and 14 NADPH for RED (this study, data not shown). However, for strain M145, only a small fraction of the carbon was used for synthesis of TBP and RED in the time period after growth cessation (Table 2). For the phosphate limited reference cultivation, e.g. ferm. #18a, only 2% (in total calculated in Cmol basis) of the carbon was used for TBP and RED. During this period of the cultivation, the L-glutamate consumption was four times higher than the D-glucose consumption (calculated on Cmol basis). The obtained yield is far from the theoretical maximal yield, between 0.4 to 0.59 on a Cmol basis for actinorhodin dependent on carbon source [39], and what has been obtained in other *Streptomyces* cultivations. For example, a *Streptomyces lividans* strain over-expressing the actinorhodin pathway specific activator *act*II-ORF4 produced actinorhodin with a yield of 15% on Cmol basis from D-glucose as sole carbon and energy source [39].

**Table 2:** Carbon consumption and derived secondary metabolite production yields in the time period 45–50 hrs

Ferm. #	Carbon source consumption		Yields [%Cmol]		
	D-glucose	L-glutamate	RED	γ-Act	TBP
	Cmol/L-h	Cmol/L-h	Cmol RED/Cmol C source	Cmol ACT/Cmol C source	Cmol TBP/Cmol C source
18a	0.0059	0.0238	1.0	0.4	1.0
19a	0.0083	0	0.1	2.2	4.9

Carbon consumption and derived secondary metabolite production yields determined for one representative cultivation each of experiments with high resolution time-series sampling for full-scale ‘omics analysis using the fully developed media and conditions for studying the effect of phosphate (ferm. #18a) and L-glutamate depletion (ferm. #19a), respectively, on metabolic switching by strain M145. Results from ‘omics analyses partly presented earlier [19-22] or to be published elsewhere.

### Analysis of ^13^ C enrichment from ^13^ C_6_ -labelled D-glucose reveals a bisection of central carbon metabolism

In order to investigate the distribution of carbon from the two carbon sources (D-glucose, L-glutamate) present in medium SSBM-P, we performed small scale cultivation experiments (200 mL) in custom made fully controlled and monitored mini-fermentors. Two cultivations were performed in parallel, supplemented with D-glucose (ferm. #24) and D-^13^ C_6_-glucose (ferm. #25), respectively. The total amount of D-glucose was reduced to 20 g/L, which, based on previous results, we expected to remain in excess until the end of the cultivation runs. A standard 1.8 L cultivation on SSBM-P medium was used as a reference (ferm. #23). The cultivation results are summarized in Figure 3A. Respiration profiles of the two small-scale cultivations were very similar both compared to each other, but also compared to the reference fermentation. The biomass concentrations obtained in ferm. #24 and #25 were above 6 g/L providing sufficient biomass for repeated sampling for metabolite analysis at different time points of cultivation. The specific productivities of TBP and RED were similar in the two 200 mL fermentations, though the initial specific TBP productivity was slightly higher than in the 1.8 L reference cultivation. Interestingly, the concentration of γ-actinorhodin, one of the different actinorhodins produced by *S. coelicolor* A3(2) and secreted into the medium [27], was almost undetectably low in the small-scale cultivations, compared to the high total amounts of TBP produced. The reason for that remains unclear. From ferm. #24 and #25, samples for intracellular metabolite analysis were withdrawn 28 h and 44 h after inoculation and processed for GC-MS analysis with subsequent calculation of Summed Fractional Labelling (SFL, % scale) of selected intracellular metabolite pools as described in the Materials and Methods section with a total of four re-samplings per time point. The labelling enrichment from ^13^ C_6_-glucose is calculated from measurements of free soluble intracellular metabolites and not protein-bound amino acids as former labelling studies in secondary metabolite producing *Streptomyces* species have used [40,41]. Hence, this study provides an instant picture of intracellular labelling enrichment at the time of sampling and not the accumulation of ^13^ C in cellular components over time. The results are given in Figure 3B and indicate a much higher labelling enrichment in the upper part of the glycolytic pathway and the pentose phosphate pathway (PPP). Interestingly, the relative labelling enrichment decreases in the PPP after growth cessation, implying that L-glutamate becomes an even more preferred carbon source during secondary metabolite production phase. Only small amounts of ^13^ C labelled carbon is found among TCA cycle metabolites which shows that the TCA cycle is heavily dominated by glutamate carbon.

## Discussion

The aim of the present study was to develop a cultivation system for systems scale studies of metabolic switching in *Streptomyces coelicolor* A3(2). This task required the simultaneous observation of several constraints provided by three considerations. (i) The scientific question itself, i.e. batch cultivations with a defined transition phase in response to the depletion of one defined nutrient and a good production phenotype. (ii) The different downstream - ‘omics - analyses and subsequent modelling approaches, i.e. a high degree of reproducibility, with biomass concentrations of >4 g/L CDW available long before nutrient depletion and an absence of analysis-interfering media components. (iii) The growth aspects of the research object *S. coelicolor* A3(2) strain M145 in submerged culture, i.e. intra-mycelial nutrient and oxygen supply and the influence of shear forces. The described approach finally led to generic conditions and two cultivation media: SSBM-P for studying metabolic switching in response to phosphate depletion, and SSBM-E which can be used for studying metabolic switching events initiated from the depletion of L-glutamate in the medium. The high reproducibility in biological replicas as demonstrated for the optimized cultivation system and the two different triggering conditions is crucial for systems scale studies of metabolic switching in *S. coelicolor* A3(2). Our results, using strain M145, imply that DO levels may influence the cultivation reproducibility. The best reproducibility of antibiotic productivities was obtained by providing a minimum of 50% DO by automatic adjustment of stirrer speed.

As demonstrated using SSBM-P and SSBM-E, respectively, including several biological replicas for high time resolution sampling for transcriptome, proteome and metabolome analysis, the refined cultivation system applying 50% DO also fulfilled the requirement of good reproducibility in full-scale experiments. Different aspects of metabolic switching have already been studied in detail and published by us based on the cultivations and respective fermentation data included in the present study [19-25]. These studies confirm the overall high reproducibility between biological replicas cultivated using the optimized system presented here and the quality of derived time-course samples for analyses on the molecular levels of global gene expression (transcriptome analysis), translation (proteome analysis) and intracellular metabolite pools (metabolite profiling), including strain M145 as well as different mutant strains. At this time, several further studies are being summarized that built up on the fundamental results of this study. Results from such studies will further unravel the complex interplay of molecular events during metabolic switching in strain M145 and its derivatives. An integrated molecular study of the effect of phosphate depletion using medium SSBM-P will for example allow for a global analysis of effects from the documented complex crosstalk of central regulators like PhoP, GlnR and AfsR [42,43] and its involvement in antibiotic production. SSBM-E may be applied to study combined molecular responses of nitrogen and carbon depletion, possibly triggering a more general stress response. However, the limiting component in this medium, L-glutamate, can obviously serve as both a carbon and nitrogen source as indicated by growth on L-glutamate alone both in (i) shake flask experiments (see Additional file 1: Table S1) and (ii) 1 L batch fermentation (ferm. #22, Figures 1 and 3C), as well as (iii) by ^13^ C distribution given in Figure 3B where carbon from L-glutamate was detected in all analysed metabolites of the central carbon metabolism. On the other hand, ammonium, accumulating in the medium during growth on L-glutamate (fermentations #19a-c in Additional file 1: Figure S1) and a possible alternative nitrogen source to L-glutamate, did not maintain growth after L-glutamate depletion (Figure 2), and cultivation on D-glucose and ammonium did not promote growth in shake flasks ( Additional file 1: Table S1) and batch fermentation (ferm. #21; Figures 1 and 3C). However, in the latter, growth could be triggered when L-glutamate was added after 80 h of cultivation (see fermentation #21 in Additional file 1: Figure S1). Therefore, if it was desired to study separate depletion of nitrogen and carbon at a systems scale, further media development would be needed. However, this would require extensive additional efforts and possibly an alternative approach of media development than the one applied in the present study.

The most striking result of the present study, however, is the complex interplay of the two sources of carbon and energy, D-glucose and L-glutamate, which need to be present to ensure a sufficiently high growth rate to prevent an early start of antibiotic production prior to either phosphate or L-glutamate depletion. When strain M145 was grown on medium SSBM-P with both carbon sources in excess, after depletion of phosphate at a cell mass of >4 g/L CDW, production of first prodiginines and subsequently actinorhodins was triggered. When D-glucose was excluded and L-glutamate remained the sole carbon source in the medium, culture growth was slightly slower, likely in response to the additional metabolic burden of the need for complete gluconeogenesis to provide precursors for cell wall synthesis and pentoses for nucleic acid biosynthesis. However, the reduction in growth rate was obviously sufficient to trigger production of blue pigmented secondary metabolites at a premature stage, simultaneously with biomass accumulation and prior to the main triggering event of phosphate depletion. Interestingly, only TBP and not RED production was triggered prematurely. This may be explained by the RED production being sensitive to phosphate repression, thus still being dependent on phosphate reduction in addition to a reduced growth rate, as also observed for a number of other antibiotics [31,32].

When the ratio of L-glutamate and phosphate in SSBM-P was altered in a way that when L-glutamate was depleted phosphate stayed in excess (as provided by SSBM-E), culture growth stopped immediately, reducing the carbon dioxide evolution rate abruptly to metabolic maintenance alone. L-glutamate appears to be both the preferred source of nitrogen and carbon, preferred in that sense that alternatives exist for both at the time of L-glutamate depletion: D-glucose was provided in excess at any time during the fermentation, and ammonium has been shown to accumulate during growth phase to significant amounts (fermentations #19a-c in Additional file 1: Figure S1). The collapse in carbon dioxide production after L-glutamate depletion implies that L-glutamate catabolism provides the main source of energy for biomass accumulation. If solely D-glucose was provided from the beginning as the sole carbon source in combination with inorganic nitrogen provided as ammonium salts, no significant culture growth could be detected in the fermentors.

Growth experiments in the presence of L-glutamate and ^13^ C_6_-D-glucose, and subsequent metabolite analysis revealed carbon from L-glutamate in significant amounts in metabolites derived from intermediates of glycolysis. In turn, some D-glucose derived carbon was found in metabolites closely linked to the TCA cycle, but at considerably lower concentrations than from L-glutamate.

These key results from labelling and cultivation experiments allow for speculations about the function of the central carbon metabolism in strain M145. When both D-glucose and L-glutamate are provided in excess, carbon flow from D-glucose through the Embden-Meyerhof-Parnas pathway and the hexose monophosphate shunt/pentose phosphate pathway (PPP), and L-glutamate through the TCA cycle both result in the production of pyruvate. L-glutamate is catabolised via α-ketoglutarate following deamination and secretion of ammonium ions into the medium, the α-ketoglutarate is decarboxylated to malate, via the TCA cycle, which in turn is decarboxylated to pyruvate via malic enzyme (malate dehydrogenase, decarboxylating; Omara, W. A. M. and Hodgson, D. A., manuscript in preparation). Catabolism of L-glutamate to pyruvate generates ten ATP molecules (presuming complete oxidation of reduced nucleotides). Catabolism of D-glucose via the Emden-Meyerhof-Parnas pathway to two molecules of pyruvate yields seven ATP molecules. Pyruvate must be subsequently decarboxylated via pyruvate dehydrogenase to acetyl-CoA, the precursor of fatty acid production and for both actinorhodin and prodiginine production.

When phosphate was depleted from SSBM-P, the continued presence of both carbon sources ensured the production of actinorhodins from acetyl CoA and also prodiginines with their more complex precursor supply pattern based on acetyl-CoA, malonyl-CoA, proline, serine, glycine and S-adenosyl methionine.

In the fermentation on SSBM-E, L-glutamate depletion after 35 hours caused the collapse in the carbon dioxide evolution rate as a consequence of growth cessation, clearly indicating that the entrance of carbon from D-glucose into the TCA-cycle in the absence of L-glutamate is largely impaired. Although L-glutamate was also used as the source of nitrogen, nitrogen in the form of increasing amounts of ammonium remained in the medium, resulting from L-glutamate catabolism. Therefore, the cessation of biomass accumulation upon L-glutamate exhaustion in SSBM-E medium, was probably due to a number of factors: concomitant exhaustion of a preferred carbon and energy source; exhaustion of a preferred nitrogen source; and a stringent response due to the nutrient down from use of amino acid to that of ammonium as nitrogen source. *S. coelicolor* A3(2) has been shown to have a classic stringent response [30]. After L-glutamate depletion, production of secondary metabolites was still triggered, however, absolute levels were comparably low to those seen during phosphate depletion. The production was also dominated by actinorhodins, which indicated that carbon flow to acetyl-CoA must have been maintained, while the production of RED was still subject to phosphate repression, as discussed above.

Therefore, we need to explain why carbon dioxide production abruptly decreases following L-glutamate exhaustion and why D-glucose as sole carbon and energy source failed to support growth. A common explanation could be a metabolic bottle neck where pyruvate accumulation results from inefficient conversion to acetyl-CoA by pyruvate dehydrogenase (PDH). Pyruvate accumulation would inhibit cell growth via the weak acid effect causing a drop in intracellular pH [44]. Surowitz and Pfister (1985) previously demonstrated that toxic accumulation of pyruvate during growth of *Streptomyces alboniger* on D-glucose was due to an imbalance between the efficiencies of glycolysis and the TCA cycle [45]. The two pathways could be balanced by the addition of adenine.

The genes characterised as encoding potential pyruvate dehydrogenase components are: SCO2183 *aceE1* pyruvate dehydrogenase (PDH) E1 component (EC 1.2.4.1); SCO2181 *aceF1* dihydrolipoyllysine-residue acetyltransferase PDH E2 component (EC 2.3.1.12); SCO2180 *lpd1* dihydrolipoamide dehydrogenase PDH E3 component (EC 1.8.1.4); SCO7124 *aceE3* PDH E1 component (EC 1.2.4.1); SCO7123 *aceF3* PDH E2 component (EC 2.3.1.12); SCO2371 *aceE2* PDH E1 component (EC 1.2.4.1); SCO1268 *aceF4* PDH E2 component (EC 2.3.1.12); SCO1269 *aceEA* PDH E1 β subunit (EC 1.2.4.1); SCO1270 *aceEB* PDH E1 α subunit (EC 1.2.4.1); SCO0884 *lpd2* dihydrolipoamide dehydrogenases E3 component (EC 1.8.1.4); SCO4919 *lpd3* dihydrolipoamide dehydrogenase E3 component (EC 1.8.1.4). The *lpd2* and *lpd3* products could potentially interact with the glycine dehydrogenase (decarboxylating) complex and brancheD-chain α-ketoacid dehydrogenase complex, however, *S. coelicolor* does not encode a lipoamide-dependent 2-oxoglutarate dehydrogenase complex. We studied the expression of the strain's pyruvate dehydrogenase (PDH) gene clusters during growth on a variety of carbon sources ( Additional file 1: Figure S2). All of the PDH clusters were found to be expressed at a constitutive level. Expression of *aceE1*, *aceF1*, *lpd1* and *lpd3* was strongly induced on alanine which is equivalent to growing on pyruvate, when deaminated. These four genes were only mildly induced on glutamate but not induced on glucose or glucose plus glutamate. The fact that growth on pyruvate necessarily requires PDH gene expression but growth on glucose does not induce expression of the pyruvate-inducible PDH supports the proposal of a metabolic bottleneck at the stage of PDH.

Growth on L-glutamate as sole carbon source was almost as good as on both carbon sources, even though both are catabolised to pyruvate. The difference between growth on L-glutamate and growth on D-glucose is that growth on the former yields ten molecules of ATP per molecule of pyruvate produced, whereas growth on the sugar only yields 3.5 molecules of ATP per molecule of pyruvate produced. In addition, when catabolising L-glutamate alone some pyruvate will be used in gluconeogenesis, reducing the pyruvate burden for the cell. This proposed pyruvate bottleneck is consistent with the results from the labelling experiment. We clearly observe that not all carbon in PPP is derived from D-glucose which shows a very flexible usage of the two carbon sources when taken up by the cell. Interestingly, the significant proportion of carbon from L-glutamate in the PPP increases further from 22% to 40% from the active growth phase to the secondary metabolite production phase, respectively.

By growing on both D-glucose and L-glutamate in SSBM-P, as in SSBM-E prior to glutamate depletion, metabolism was characterized by a complex interplay of carbon flow from both carbon sources. L-glutamate for ATP generation and acetyl-CoA for antibiotic production were used efficiently even in the presence of high amounts of D-glucose. By that means, the bacterium’s energy and reducing power needs were provided mainly by L-glutamate catabolism with a supporting role of D-glucose. This ensures rapid growth, a rapid accumulation of biomass, the prevention of a premature triggering of and - after metabolic transition - an efficient production of secondary metabolites.

## Conclusions

The present study presents for the first time the development of a dedicated cultivation strategy including cultivation conditions and two fully defined media fulfilling the requirements for systems biology studies of metabolic switching in *S. coelicolor* A3(2) in response to the depletion of phosphate and L-glutamate, respectively. Key challenges addressed using solely defined media components were: (i) a high degree of reproducibility between biological replicas, (ii) a defined transition phase in response to one defined nutrient depletion event, as well as (iii) sufficiently high concentrations of biomass already long before this transition triggering event. The best reproducibility was obtained by providing a minimum of 50% DO by automatic adjustment of stirrer speed. Prevention of premature triggering of secondary metabolite production and sufficiently high biomass concentrations prior to nutrient depletion were achieved by providing L-glutamate in combination with excess D-glucose in both media described, SSBM-P and SSBM-E.

Labelling and cultivation experiments on either or both of the two carbon sources were performed to understand why both carbon sources were necessary to fulfil the requirements for systems scale studies. The results revealed a complex interplay of carbon from both sources in the bacterium's central carbon metabolism. In the presence of D-glucose, L-glutamate was found to act as the preferred carbon source, while D-glucose alone appeared incapable of maintaining culture growth, likely due to a metabolic bottleneck at the oxidation of pyruvate to acetyl-CoA.

## **Competing interests**

The author(s) declare that they have no competing interests related to the presented work.

## Authors’ contributions

The main contributions of the different authors to the presented work were as follows: AW participated in drafting the study and performing all cultivations, conducted the metabolite analysis and drafted the manuscript. PB participated in performing the metabolite analysis, analysing its results and drafting the manuscript. AØ participated in cultivation experiments. ØMJ participated in drafting the study and performing cultivation experiments. HS participated in drafting the study. WAMO performed the gene expression analysis of PDH genes clusters. DAH provided an interpretation of results and participated in finalizing the manuscript. TEE participated in drafting and performing the study, the interpretation of results and finalizing the manuscript. All authors read and approved the final manuscript version.

## Supplementary Material

Additional file 1 Table S1. Combinations of carbon and nitrogen sources tested in shake flask experiments. Growth was followed by measuring OD_450_, production was followed by visual inspection. Growth phenotype: + fast growth with no/minor lag phase, fast biomass built-up, high biomass concentration; - slow growth and/or long lag phase and/or low biomass yield; -- no/insignificant growth. Production phenotype: + good production yield of red and blue pigments with start after biomass built-up, - low production yield and/or early start of production at low OD. Additional file 1: Figure S1. On-line and off-line measurements as a function of time for all cultivations included in the present study (see Figure 1 for details on the media and cultivation conditions). DO, dissolved oxygen [%]; RPM, agitation [rpm]; CO_2_, CO_2_ evolution rate [mmoL/L/h]; CDW, cell dry weight [g/L]; ACT/RED/TBP, γ-actinorhodin/undecylprodigiosin/total blue pigments [spec. units]; PO4, medium phosphate [mg/L]; Glu/Glc, L-glutamate/D-glucose in the medium [g/L]; NH4+, ammonium [mg/L], values compensated for cross reaction of the assay with L-glutamate in the medium. Values have, where applicable, been multiplied with or divided by factors as given behind the respective legend entry in order to fit all data on one common axis. Additional file 1: Figure S2. Expression of the strain M145 pyruvate dehydrogenase (PDH) gene clusters during growth on a different carbon sources. Data were handled as described in Nieselt *et al.*[19] using GeneSpring®. Click here for file
