# Supplementary material for: Optimized submerged batch fermentation strategy for systems scale studies of metabolic switching in Streptomyces coelicolor A3(2)
Source: BMC Syst Biol. 2012 Jun 7;6:59. doi: 10.1186/1752-0509-6-59 (PMC3431225; doi:10.1186/1752-0509-6-59)
Supplement: Additional file 1 — Table S1. Combinations of carbon and nitrogen sources tested in shake flask experiments. Growth was followed by measuring OD450, production was followed by visual inspection. Growth phenotype: + fast growth with no/minor lag phase, fast biomass built-up, high biomass concentration; - slow growth and/or long lag phase and/or low biomass yield; -- no/insignificant growth. Production phenotype: + good production yield of red and blue pigments with start after biomass built-up, - low production yield and/or early start of production at low OD. Additional file 1: Figure S1. On-line and off-line measurements as a function of time for all cultivations included in the present study (see Figure 1 for details on the media and cultivation conditions). DO, dissolved oxygen [%]; RPM, agitation [rpm]; CO2, CO2 evolution rate [mmoL/L/h]; CDW, cell dry weight [g/L]; ACT/RED/TBP, γ-actinorhodin/undecylprodigiosin/total blue pigments [spec. units]; PO4, medium phosphate [mg/L]; Glu/Glc, L-glutamate/D-glucose in the medium [g/L]; NH4+, ammonium [mg/L], values compensated for cross reaction of the assay with L-glutamate in the medium. Values have, where applicable, been multiplied with or divided by factors as given behind the respective legend entry in order to fit all data on one common axis. Additional file 1: Figure S2. Expression of the strain M145 pyruvate dehydrogenase (PDH) gene clusters during growth on a different carbon sources. Data were handled as described in Nieselt et al.[19] using GeneSpring®. [file 1752-0509-6-59-S1.pdf]

Carbon source(s)	Nitrogen source(s)	Growth phenotype	Production phenotype
L-Glutamate	$NH4^+$, L-glutamate	+	-
D-Glucose	$NH4^+$	-	-
Glycerol	$NH4^+$	-	-
α-Ketoglutarate	$NH4^+$	-	-
D-Mannitol	$NH4^+$	--	-
Acetate	$NH4^+$	--	-
Citrate	$NH4^+$	--	-
Pyruvate	$NH4^+$	--	-
Succinate	$NH4^+$	-	-
D-Fructose	$NH4^+$	--	-
L-Arabinose	$NH4^+$	-	-
D-Galactose	$NH4^+$	--	-
L-Glutamate, D-Glucose	L-Glutamate	+	+
L-Glutamate	L-Glutamate	+	-
D-Glucose, glycerol	$NH4^+$	-	-
D-Glucose, α-ketoglutarate	$NH4^+$	--	-
D-Glucose, succinate	$NH4^+$	--	-
D-Glucose, L-glutamate	$NH4^+$, L-glutamate	+	+
D-Glucose, mannitol	$NH4^+$	--	-
D-Glucose, arabinose	$NH4^+$	-	+
Glycerol, α-ketoglutarate	$NH4^+$	-	-
Glycerol, L-glutamate	L-Glutamate	+	+
α-Ketoglutarate, L-glutamate	L-Glutamate	+	-
D-Glucose	Urea	-	-
D-Glucose, α-ketoglutarate	Urea	--	-
D-Glucose, casamino acids	$NH4^+$, casamino acids	+	+

WENTZEL *et al.* - SUPPLEMENTARY TABLE ST1

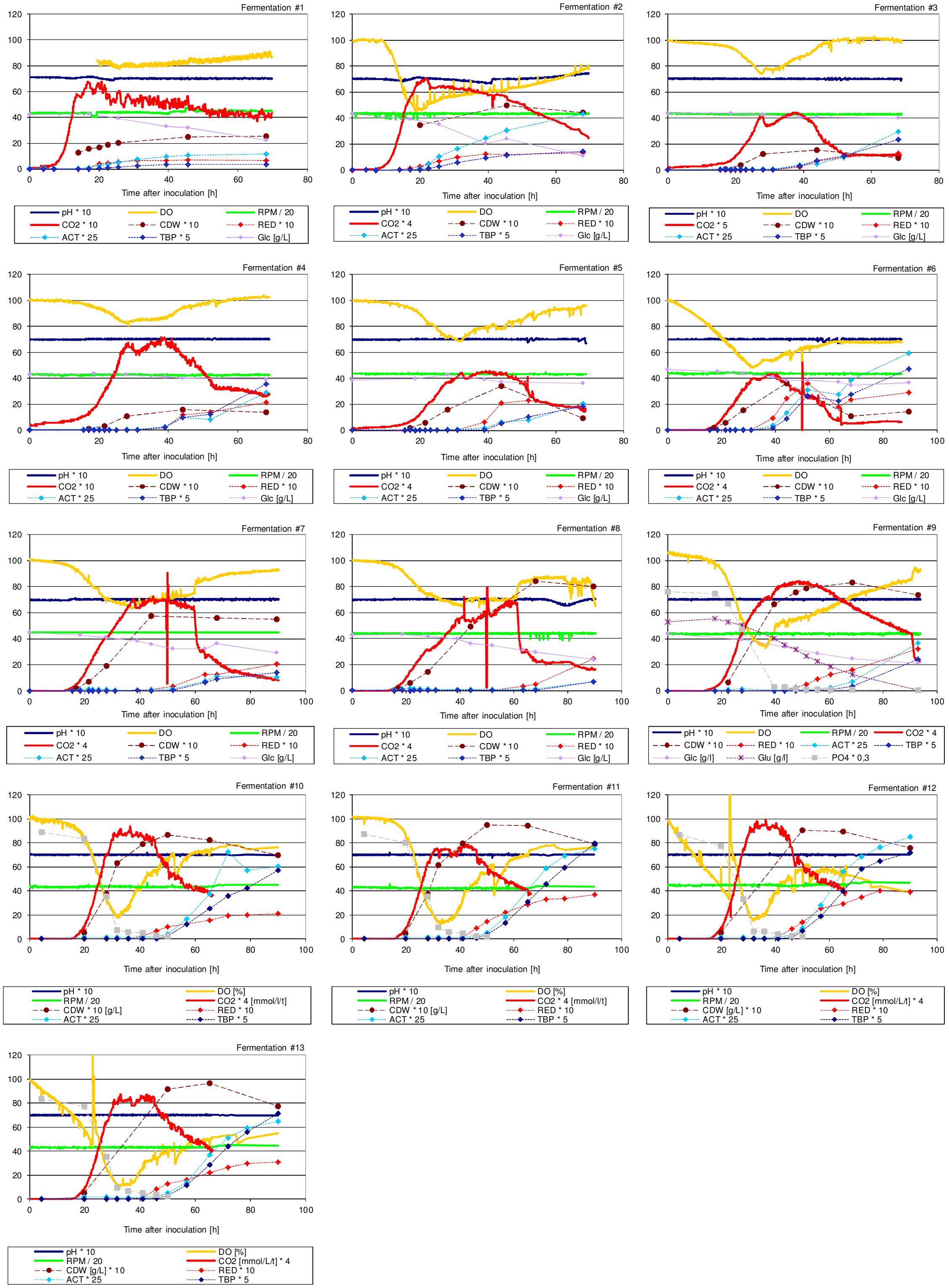

WENTZEL *et al.* – SUPPLEMENTARY FIGURE SF1 (part 1 of 3)

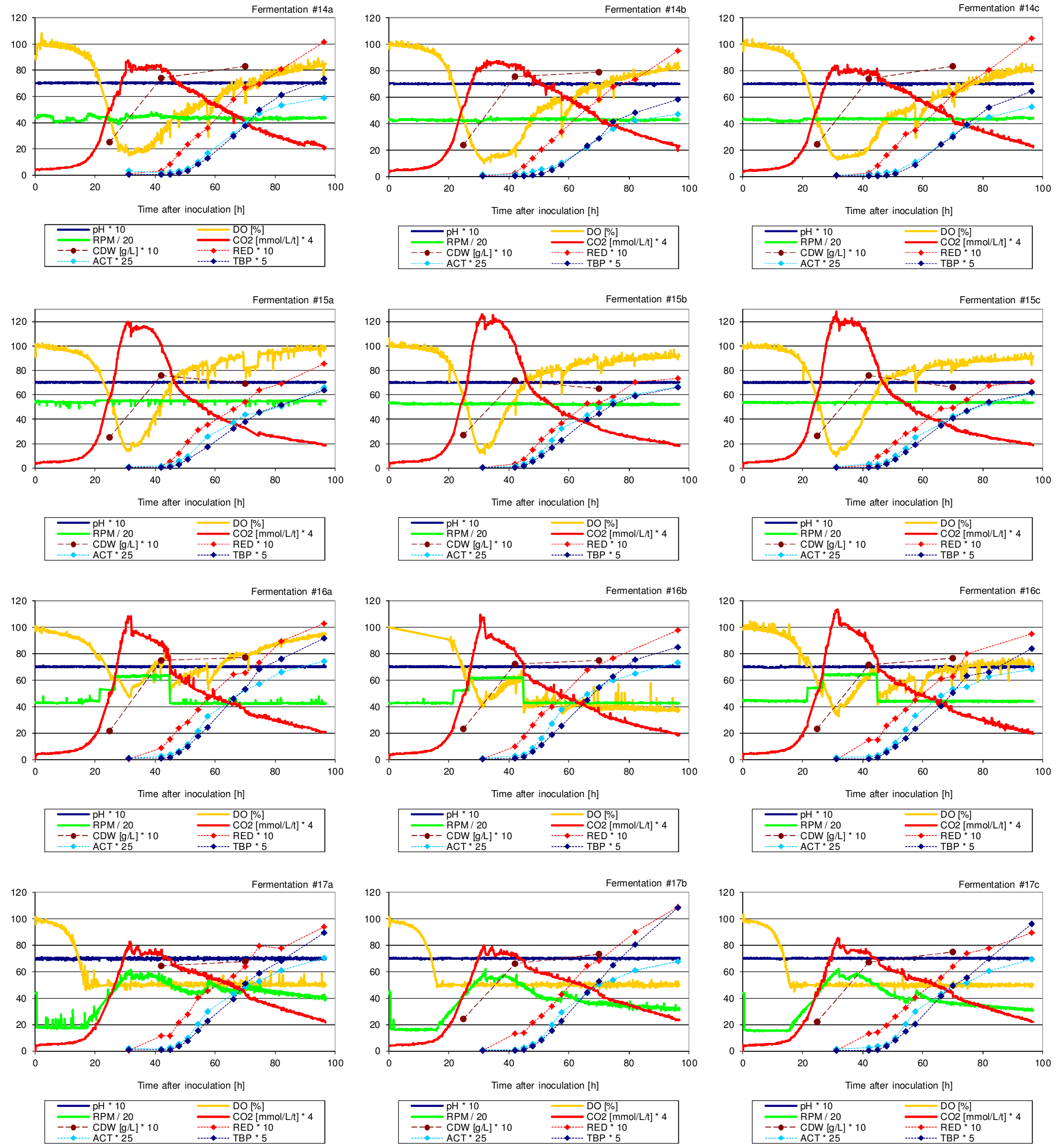

WENTZEL *et al.* – SUPPLEMENTARY FIGURE SF1 (part 2 of 3)

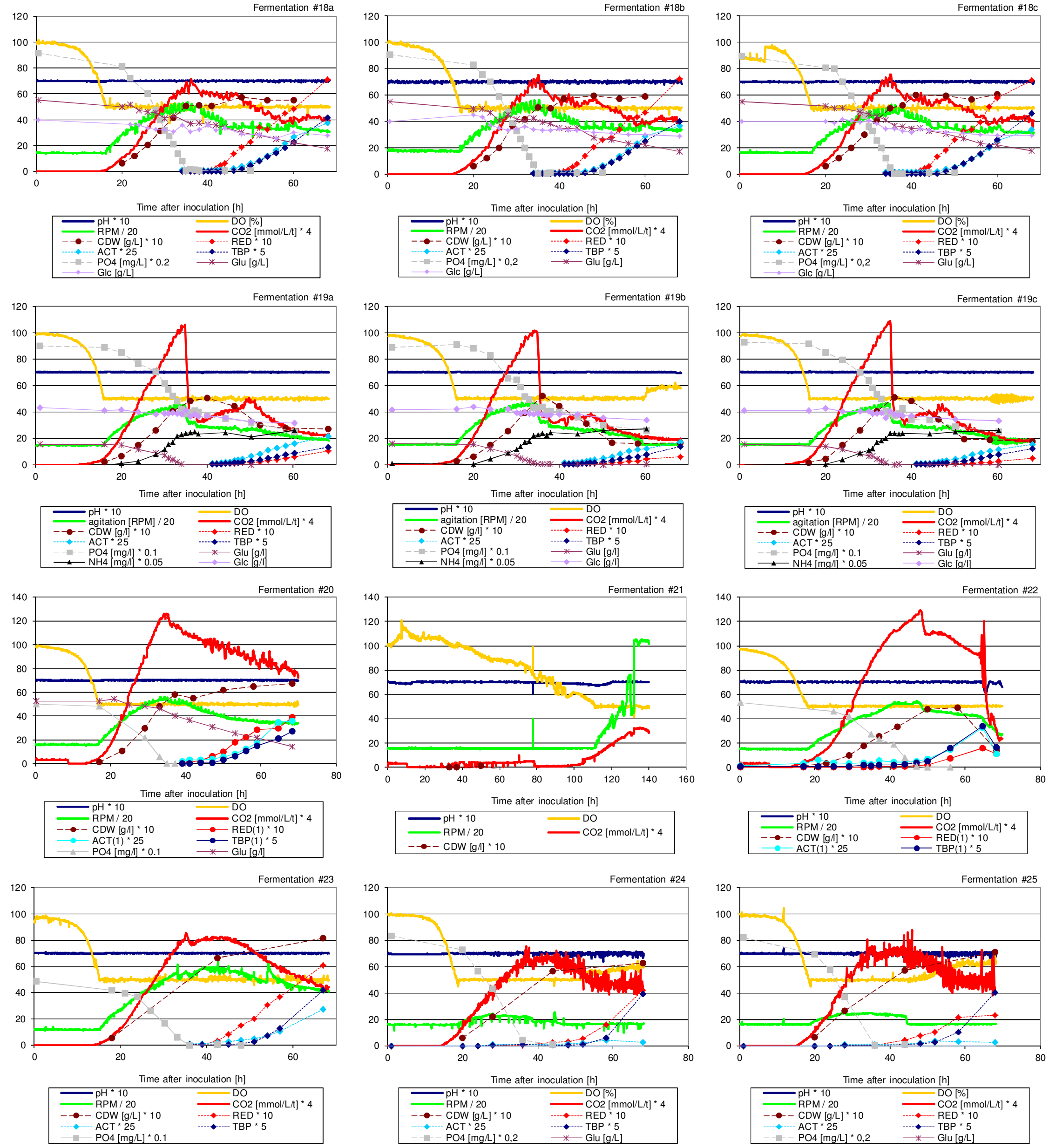

WENTZEL *et al.* – SUPPLEMENTARY FIGURE SF1 (part 3 of 3)

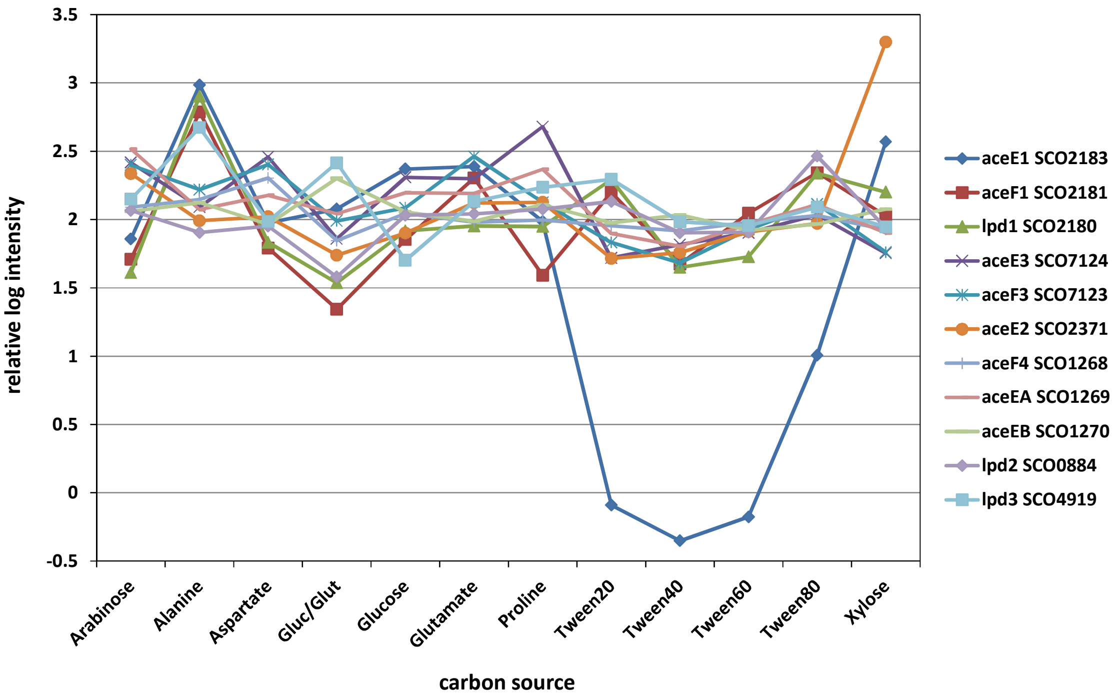

WENTZEL *et al.* – SUPPLEMENTARY FIGURE SF2
